# Assessment of PABPN1 nuclear inclusions on a large cohort of patients and in a human xenograft model of oculopharyngeal muscular dystrophy

**DOI:** 10.1007/s00401-022-02503-7

**Published:** 2022-10-05

**Authors:** Fanny Roth, Jamila Dhiab, Alexis Boulinguiez, Hadidja-Rose Mouigni, Saskia Lassche, Elisa Negroni, Laura Muraine, Alix Marhic, Alison Oliver, Jeanne Lainé, Andrée Rouche, Erin K. O’Ferrall, Baziel van Engelen, Coen Ottenheijm, Hagar Greif, Sergiu Blumen, Jean Lacau St Guily, Sophie Perie, Gillian Butler-Browne, Vincent Mouly, Capucine Trollet

**Affiliations:** 1 Myology Center for Research, U974, Sorbonne Université, INSERM, AIM, GH Pitie Salpetrière Bat Babinski, 47 bd de l’Hopital, 75013 Paris, France; 2 Department of Physiology, Amsterdam University Medical Center, Amsterdam, The Netherlands; 3 Department of Neurology and Neurosurgery, McGill University, Montréal, Canada; 4 Department of Pathology, Montreal Neurological Institute and Hospital, McGill University, Montréal, Canada; 5 Department of Neurology, Donders Institute for Brain, Cognition and Behaviour, Radboud University Medical Center, Nijmegen, The Netherlands; 6 BioBlast-Pharma Ltd., Tel-Aviv, Israel; 7 Department of Neurology, Hadera and Rappaport Faculty of Medicine, Hillel Yaffe Medical Center, The Technion, 1 Efron Street, 31096 Haifa, Israel; 8 Department of Otolaryngology-Head and Neck Surgery, Faculty of Medicine and Sorbonne, Université, Tenon Hospital, APHP, Paris, France; 9 Department of Otolaryngology-Head and Neck Surgery, Rothschild Foundation Hospital and Sorbonne University, Paris, France; 10 Department of Otolaryngology Head and Neck Surgery, Com Maillot-Hartmann Clinic, Neuilly-sur-Seine, France

**Keywords:** Nuclear aggregates, Inclusions, PABPN1, OPMD, Human biopsies, Loss of function, Xenograft

## Abstract

Oculopharyngeal muscular dystrophy (OPMD) is a rare muscle disease characterized by an onset of weakness in the pharyngeal and eyelid muscles. The disease is caused by the extension of a polyalanine tract in the Poly(A) Binding Protein Nuclear 1 (PABPN1) protein leading to the formation of intranuclear inclusions or aggregates in the muscle of OPMD patients. Despite numerous studies stressing the deleterious role of nuclear inclusions in cellular and animal OPMD models, their exact contribution to human disease is still unclear. In this study, we used a large and unique collection of human muscle biopsy samples to perform an in-depth analysis of PABPN1 aggregates in relation to age, genotype and muscle status with the final aim to improve our understanding of OPMD physiopathology. Here we demonstrate that age and genotype influence PABPN1 aggregates: the percentage of myonuclei containing PABPN1 aggregates increases with age and the chaperone HSP70 co-localize more frequently with PABPN1 aggregates with a larger polyalanine tract. In addition to the previously described PRMT1 and HSP70 co-factors, we identified new components of PABPN1 aggregates including GRP78/BiP, RPL24 and p62. We also observed that myonuclei containing aggregates are larger than myonuclei without. When comparing two muscles from the same patient, a similar amount of aggregates is observed in different muscles, except for the pharyngeal muscle where fewer aggregates are observed. This could be due to the peculiar nature of this muscle which has a low level of PAPBN1 and contains regenerating fibers. To confirm the fate of PABPN1 aggregates in a regenerating muscle, we generated a xenograft model by transplanting human OPMD muscle biopsy samples into the hindlimb of an immunodeficient mouse. Xenografts from subjects with OPMD displayed regeneration of human myofibers and PABPN1 aggregates were rapidly present—although to a lower extent-after muscle fiber regeneration. Our data obtained on human OPMD samples add support to the dual non-exclusive models in OPMD combining toxic PABPN1 intranuclear inclusions together with PABPN1 loss of function which altogether result in this late-onset and muscle selective disease.

**Supplementary Information:**

The online version contains supplementary material available at 10.1007/s00401-022-02503-7.

## Introduction

The misfolding of proteins and their further accumulation as insoluble amyloid aggregates are typical features of many human neurological and neuromuscular diseases, including oculopharyngeal muscular dystrophy (OPMD), a rare genetic repeat expansion disease. In OPMD, a short abnormal polyalanine expansion in the ubiquitous RNA-binding protein PABPN1 (Poly(A)-Binding Protein Nuclear 1) leads to muscle dysfunction with clinical manifestations primarily restricted to a limited group of muscles: eyelid and pharyngeal muscles are the first affected muscles leading, respectively, to ptosis and dysphagia [64]. Proximal limb muscles often become affected at later stages [68]. The wild-type PABPN1 protein contains 10 alanines whereas the mutated form can contain from 1 to 8 additional alanines [19, 33, 52]. Recently, a positive correlation was demonstrated between the size of the alanine expansion in PABPN1 and the age of diagnosis and severity of the disease [53]. This mutation in the PABPN1 protein leads to the accumulation of inclusions of tubulofilamentous aggregates in myonuclei of patient biopsy samples. These aggregates are found in every OPMD muscle and constitute the main histopathological marker of the disease [31, 66]. Many studies have hypothesized their deleterious role by associating them with defects observed in OPMD such as ubiquitin–proteasome dysfunction [2, 4], mRNA splicing impairment [36], apoptosis [27] and endoplasmic reticulum stress [41]. Moreover, it has been shown that reducing nuclear aggregates ameliorates phenotype in both cellular and animal models of OPMD [1, 9, 27, 41, 62] and a promising therapeutic approach targeting aggregates via autophagia using intravenous injection of trehalose is already in phase II clinical trial [6]. On the other hand, a recent study in drosophila pointed to a decorrelation between muscle phenotype and PABPN1 aggregation [51] and other pathological features such as impaired mitochondrial function with no direct relation to PABPN1 aggregates have been demonstrated in OPMD [21, 69]. Therefore today, the exact contribution of PABPN1 aggregates to human disease is still unclear. Since the discovery of nuclear aggregates in 1980, several studies have attempted to quantify both their size and number in OPMD patient biopsy samples [10, 15, 18, 25, 65, 66]. The first observation of intranuclear aggregates described tubular filaments with an external diameter of 8,5 nm and an internal diameter of 3 nm [66]. Additional studies showed that the volume occupied by the nuclear aggregates can be variable [18] with some aggregates occupying most of the nuclei while others were limited to less than 1% of the nuclei [65]. The percentage of nuclei containing aggregates ranged from 1 to 10% in different patient populations including French, French-Canadian and Bukharan Jews [10, 16, 18]. All of these studies were performed by electron microscopy, making an exhaustive qualitative and quantitative study fastidious and time-consuming. Moreover, OPMD being a rare disease, it has always been difficult to make a correlation with age, mutation or muscle, due to the rare availability of patient material. To gain critical knowledge on OPMD pathogenesis, we have gathered a large collection of OPMD muscle biopsy samples from several countries and used immunofluorescence to quantify the percentage of nuclei containing aggregates, as well as to determine the size and composition of the nuclear aggregates. In addition to the previously described PRMT1 and HSP70 co-factors, we identified new components of PABPN1 aggregates including GRP78/BiP, RPL24 and p62. We have studied the correlation between nuclear aggregates, nuclear size, genotype and age of the patients and using HSP70 co-staining we show for the first time that the composition of nuclear aggregates varies with the genotype of the patients. Interestingly, myonuclei containing aggregates are larger than nuclei without. We also observed less PABPN1 aggregates in the clinically affected OPMD cricopharyngeal muscle than in other muscles. This pharyngeal muscle contains less PABPN1 and regenerating fibers. To analyze the fate of PABPN1 aggregates during muscle regeneration we created a human OPMD xenograft model and confirmed the formation of PABPN1 aggregates shortly after muscle regeneration although to a lower extent than that initially present before muscle regeneration. Altogether our data help to clarify the puzzle of OPMD pathogenesis and the status of PABPN1 aggregates. Our data obtained in human samples fit with the proposed two non-mutually exclusive models combining a loss of PABPN1 function in affected muscles and a detrimental role of nuclear aggregates in the disease [8]. The variations observed with age and genotype and muscle selectivity further explain the late-onset disease, muscle-restricted and genotype/phenotype correlation observed in OPMD patients.

## Subjects/materials and methods

### Muscle biopsies

We collected 90 biopsy samples from different muscles including sternocleidomastoid (SCM), tibialis anterior (TA), vastus lateralis (VLM), deltoid (DM) and cricopharyngeal (CPM) muscles of 73 OPMD patients. Biopsy samples were collected either after myotomy for the 46 French patients or by needle biopsy for the 10 Canadian patients, the 11 Israeli patients and the 6 Dutch patients. The ages of the subjects at biopsy ranged from 43 to 83 years. All OPMD patients were genetically diagnosed: 70 were heterozygous patients (GCN)10-n (with n comprised between 11 and 17) and 3 were homozygous (GCN)11–11 or (GCN)13–13 (see Table 1 for full description). All individuals selected for the study underwent surgical procedures after informed consent in accordance with the ethical rules following the legislation of the countries where they have been operated.

**Table 1:** Age and genotype of OPMD patients

Genotype	Number of patients	Age (mean ± sem)	Biopsies from same patient		
			SCM/ CPM	SCM/DM	VLM/TAM
(GCN)10–11	1	80	/	/	/
(GCN)10–12	4	73.8 ± 8.8	/	/	/
(GCN)10–13	41	60.9 ± 9.3	2	1	/
(GCN)10–14	8	67.1 ± 7.9	/	1	/
(GCN)10–15	11	59.7 ± 10.6	5	1	1
(GCN)10–16	4	51.8 ± 7	/	/	3
(GCN)10–17	1	63	/	/	/
(GCN)11–11	2	67.5 ± 0.7	1	1	/
(GCN)13–13	1	47	1	/	/

Biopsy samples from OPMD patients have been collected in 4 different countries: France, Canada, Israel and The Netherlands. While most of the patients are heterozygous with a normal allele (GCN)10 and a mutated allele (GGN)n with n representing the number of (GCN) triplet expansion ranging from 11 to 17, 3 are homozygous (11–11 or 13–13). The number of patients for which several muscles have been biopsied is listed in the 3 last columns of the table

*SCM* sternocleidomastoid muscle, *CPM* cricopharyngeal muscle, *DM* deltoid muscle, *VLM* vastus lateralis muscle, *TAM* tibialis anterior muscle

### Sample preparation

Muscle biopsy samples were mounted on tragacanth gum (6% in water; Sigma-Aldrich, St Louis, MO), frozen in isopentane precooled in liquid nitrogen and stored at − 80 °C. For histological and immunofluorescent analyses, biopsy samples were cut into 5 µm cryosections using a cryostat (Leica CM1850) and collected on glass Superfrost plus slides (Menzel Gläser, J1800AMNZ, Thermo Scientific). To assess tissue morphology, the sections were stained with Hematoxylin and Eosin. Structural abnormalities were assessed by Gomori trichrome staining.

### Immunofluorescence

Immunostaining of muscle biopsy samples was performed as previously described [31]. Briefly, muscle sections were thawed at room temperature and then fixed in 100% cold acetone for 10 min. In order to reveal only the proteins trapped in aggregates, the muscle sections were then incubated in 1 M potassium chloride (KCl) to remove all soluble proteins. Primary antibodies against PABPN1 (ab75855 Abcam; 1:100), HSP70 (MA1-90,504 Invitrogen; 1:100), PRMT1 (ab73246 Abcam; 1:100), dystrophin (NCL-dys1 Novocastra; 1:10), laminA/C (ab40567 Abcam; 1:100), p62/SQSTM1 (MBL PM045 1:200), RPL24 (GTX114729 1:100), GRP78/BiP (CST3177 Cell signaling 1:200) were then incubated overnight at 4 °C. When necessary a directly conjugated PABPN1-488 antibody (ab206056 Abcam) was used. The next day, the muscle sections were rinsed and incubated with the appropriated fluorescent secondary antibodies prior to counterstaining nuclei with Hoechst. For the eMyHC, spectrin and laminA/C staining, slides were thawed at RT and blocked in PBS containing 2% FBS for 30 min at RT. Primary antibodies for embryonic myosin (F1.652 DSHB; 1:4), human spectrin (NCL-SPEC Novocastra; 1:50) human laminA/C (ab40567 Abcam, 1:300) and laminin (Z0097 Dako; 1:400) were incubated for 1 h at RT, sections were rinsed and incubated with appropriate secondary antibodies prior to counterstaining with Hoechst.

### Quantification of nuclear aggregates

The percentage of myonuclei containing nuclear aggregates in muscle biopsy samples was determined by blinded manual counting of the number of nuclei containing aggregates as related to the total number of myonuclei on the section. 500 to 1000 nuclei per muscle were counted. The quantification was repeated 2 to 5 times for all patients except for patient (GCN)10/17 where the biopsy material available allowed us to perform the immunofluorescence analysis only once. The standard error (SE) was calculated as σ/√n with σ the standard deviation (SD) for each counting and *n* the number of counting per patient. The mean standard error (SEM) represents the mean of SE measurement for each patient and corresponds to 0.8% ± 0.07.

### Quantification of nuclei and nuclear aggregate size

On a subset of biopsy samples, a laminA/C and PABPN1 co-staining was performed to delineate and quantify the respective size of the nucleus and the aggregate. The CSA of each myonuclei and each PABPN1 aggregate was measured automatically using image J.

### Western-blot

Proteins were extracted by sonicating cells in RIPA buffer 0.15 M NaCl, 0.1% SDS, 50 mM Tris (pH 8), 2 mM EDTA and 10% Triton-X-100 with protease inhibitor cocktail (Complete, Roche Diagnostics). Similar quantities of proteins were separated on 4–12% Bis–Tris gels (Invitrogen) and transferred onto a PVDF membrane (Invitrogen) for 1 h at a constant 250 mA at 4 ℃. Membranes were blocked by incubation in 5% BSA in 0.1 M TBS, 0.1% Tween-20 for 1 h at room temperature under agitation. Membranes were stained with primary antibodies directed against PABPN1 (1:1000, Abcam, ab75855) and GAPDH–HRP (1:1000, Abcam). Membranes were then incubated with appropriate secondary antibodies conjugated to HRP (except for GAPDH-HRP) and the Chemidoc system (Bio-Rad) with an ECL revelation kit (ThermoFischer, 35,050) was used to detect the signals from the membranes.

### Mouse xenotransplantation

The procedure was performed according to a protocol previously described [46, 72]. Immunodeficient mice (*Rag2*^*−/−*^*IL2Rb*^*−/−*^) were anaesthetized with an intraperitoneal injection of ketamine hydrochloride (80 mg/kg) and xylazine (10 mg/kg) (Sigma-Aldrich, St Louis, MO). Human biopsies were obtained from the MyoBank, a Biological Resource Center affiliated with EuroBioBank and with national authorization to distribute human tissues (authorization AC-2019–3502 from the French Ministry of Research) after informed consent from the patient. After removal of the *tibialis anterior* (TA) and *extensor digitorum longus* (EDL) muscles of the mouse, the fresh human muscle biopsy was ligated with a non-absorbable suture to the tendons of the *peroneus longus* muscle. Skin was closed with surgical resorbing sutures. Buprenorphine (0.1 mg/kg) was given subcutaneously before and after the surgery for pain control. Four months after grafting, the human muscle graft was collected and mounted on tragacanth gum and frozen in isopentane precooled in liquid nitrogen and kept at − 80 °C. The protocol for xenotransplantation was approved by the national ethical committee for animal experiments (approval no. 4165–2016021715164682 delivered by the French Ministry of Higher Education and Scientific Research).

### Image acquisition

Widefield fluorescence images were taken with an Olympus BX70 microscope (Olympus Optical, Hamburg, Germany) equipped with a CCD Camera (Photometrics CoolSNAP fx; Roper Scientific) and driven by Metaview (Universal Imaging, Downington, PA, USA) and with an Axio Observer 7 microscope (Zeiss) equipped with a motorized stage coupled with an Orca Flash 4 Camera (Hamamatsu), and driven by the Zen software (Zeiss). Confocal images were taken with a Nikon Ti2 microscope equipped with a motorized stage and a Yokogawa CSU-W1 spinning disk head coupled with a Prime 95 sCMOS camera (Photometrics), driven by Metamorph. Serial widefield fluorescence images were acquired using a NanoZoomer Hamamatsu Scanner. Images were then analyzed using MetaView image analysis software (Universal Imaging, Downington, PA, USA) or Imaris Microscopy Image Analysis software. Image J and Fiji were used for quantification analysis.

### Statistics

All data are mean values ± standard error of the mean. Statistical analyses were performed using the Student *t* test and the one-way Anova with the Bonferroni post-hoc analysis as indicated. GraphPad Prism (version 4.0b; GraphPad Software, San Diego California, USA) was used for the analyses. A difference was considered to be significant at *P < 0.05, **P < 0.01 or ***P < 0.001.

## Results

**Fig. 1:**
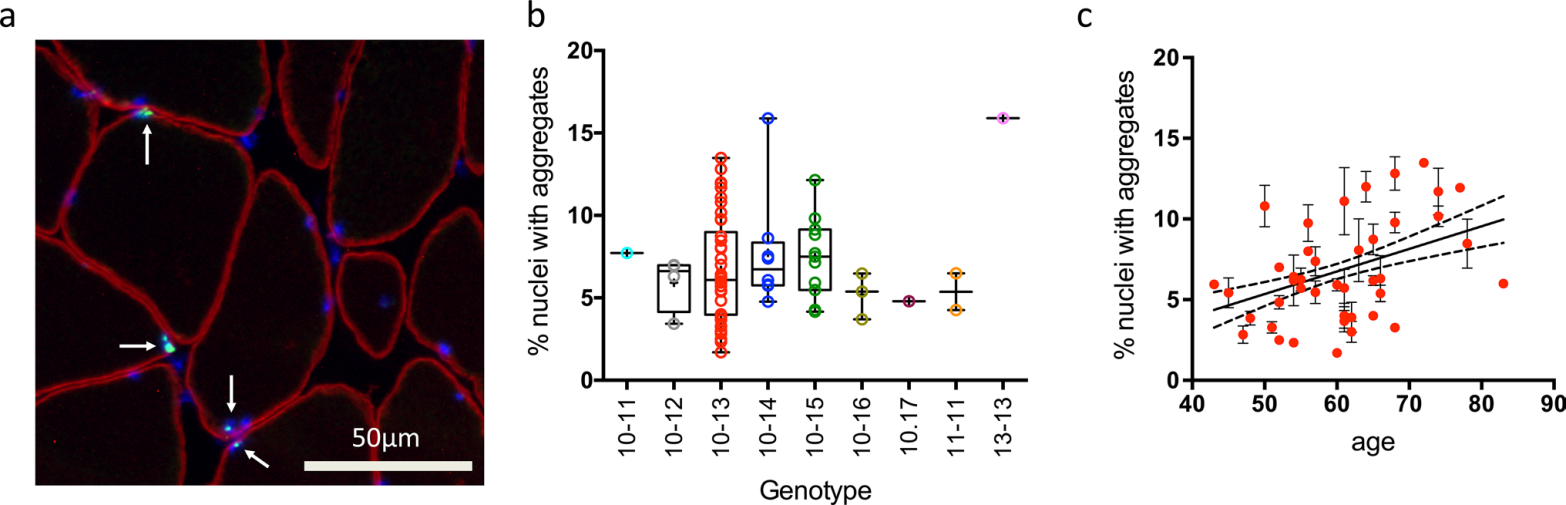
The mean percentage of nuclei containing aggregates does not vary with PABPN1 genotype but varies with age. **a** PABPN1 staining (green) performed after a KCl 1 M pre-treatment on 5 μm-thick cryosections. Nuclei are counterstained with dapi (blue) and muscle fibers delineated with an anti-dystrophin (red) staining. Scale bar = 50 µm. Aggregates are indicated with an arrow. **b** Box-plot and individual values of the mean percentage of myonuclei containing aggregates for each genotype. Within one genotype, the age of the patients is variable. Each dot represents a patient. Mean and SEM are indicated for each genotype. Muscle biopsy samples used: 10–11: SCM, 10–12: SCM, 10–13: SCM/DTM, 10–14: SCM-DTM, 10–15: SCM/DTM/TAM/VLM, 10–16: VLM/TAM, 10–17: VLM, 11–11: SCM/DTM, 13–13: SCM. No statistical difference is observed using a one-way Anova test on all the genotypes except (GCN)10–11, (GCN)10–17, (GCN)11–11 and (GCN)13–13 containing only one or two patient. **c** Correlation between age at biopsy and percentage of nuclei with aggregates for the 41 patients genotyped (GCN)10–13. Each dot represents a patient. Error bar represents experimental error on the measure. Linear regression statistic test: slope: 0.1401 ± 0.03587; R2 = 0.1080; *p* < 0.0002

In the present study, we quantified the percentage of myonuclei containing nuclear aggregates in OPMD muscle biopsies using PABPN1 immunofluorescence. We analyzed 90 frozen muscle biopsies from 73 OPMD patients with known genotype and age: 70 heterozygous patients ranging from (GCN)10–11 to (GCN)10–17 genotype and 3 homozygous patients carrying the genotype (GCN)11–11 or (GCN)13–13, all aged between 43 and 83 years old (Table 1). For each muscle biopsy, aggregates were revealed and quantified using a PABPN1 immunofluorescence staining after 1 M KCl pre-treatment to eliminate all soluble proteins (including soluble PABPN1) (Fig. 1a). All genotypes included, the percentage of nuclei containing aggregates ranged from 2 to 15% on all muscle sections including those of homozygous patients, which is consistent with previous studies using electron microscopy or fluorescence microscopy. The highest percentage of aggregates (around 15%) was observed for the homozygous (GCN)13–13 patient as well as for one (GCN)10–14 patient (Fig. 1b). When analyzing the mean percentage of nuclei containing aggregates for each heterozygous genotype (no matter the age), no significant difference among groups was observed, with on average 5–7% of myonuclei containing aggregates (Fig. 1b). In one (GCN)10–13 OPMD biopsy where a larger amount of muscle was available, we evaluated the distribution of aggregates along elongated multinucleated muscle fibers. For this sample, we cut and stained 30 consecutive serial 5 μm-cryosections. By analyzing manually the whole cross-section area (containing 220 fibers) on this 150 μm length of this muscle fragment we observed a quite homogeneous distribution of aggregates throughout the muscle fiber with more than 95% of the fibers contained at least one aggregate (Supplementary Fig. 1, online resource). Within a group of patients with the same genotype, the percentage of nuclear aggregates was very variable e.g. in the 41 heterozygous patients with the (GCN)10–13 genotype the percentage varied from 2 to 12%. To analyze a potential link between age and percentage of nuclei containing aggregates we restricted our analysis to this group of (GCN)10–13 heterozygous genotype for which we had the highest number of patients (*n* = 41). By plotting the percentage of nuclei containing aggregates versus age at biopsy, we observed that the number of aggregates was positively correlated with the age of the patient (Fig. 1c; *p* < 0,0002).

**Fig. 2:**
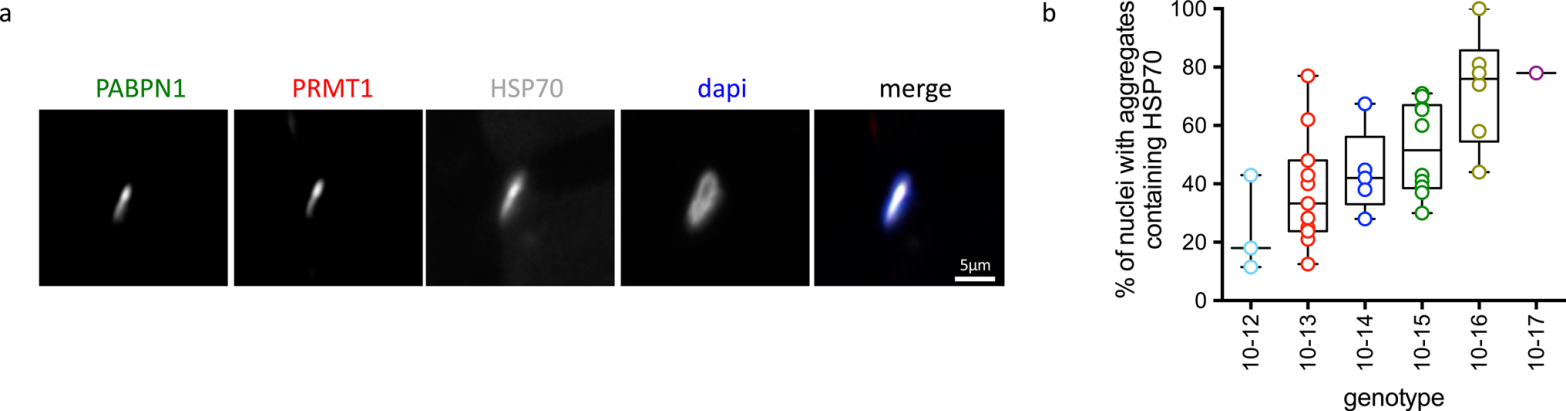
The composition of nuclear aggregates is heterogeneous. **a** HSP70 and PRMT1 co-staining in PABPN1-aggregates. **b** Percentage of nuclear aggregates containing HSP70 varies with the genotype. Muscle biopsies used: 10–12: SCM, 10–13: SCM/DTM, 10–14: SCM-DTM, 10–15: SCM/DTM/TAM/VLM, 10–16: VLM/TAM, 10–17: VLM. One-way Anova and post-test for linear trend indicates that linear trend is significant *p* < 0.001

To further investigate a potential link between the composition of nuclear aggregates and the number of expanded repeats, we analyzed the effect of the patient’s genotype on the composition of the nuclear aggregates. To do this, we selected HSP70 and PRMT1, known to be trapped in aggregates [59, 62], and performed co-staining with antibodies against PABPN1 and either HSP70 or PRMT1 (Fig. 2a). We observed different patterns of expression of these two proteins within aggregates: whereas PRMT1 was present in all PABPN1 nuclei aggregates, independently of the genotype, HSP70 was more heterogeneously distributed: patients with the largest (GCN) PABPN1 expansion contained more HSP70-positive aggregates (Fig. 2b). Since PRMT1 was present in all PABPN1 aggregates, we looked at other PRMT1 binding partners among the several candidates recently identified [70] and also confirmed the presence of the chaperone GRP78/BiP and the ribosomal protein RPL24 within some but not all aggregates (Supplementary Fig. 2, online resource). The ubiquitin-binding protein p62/SQSTM1 was also found in some but not all PABPN1 aggregates (Supplementary Fig. 2, online resource).

**Fig. 3:**
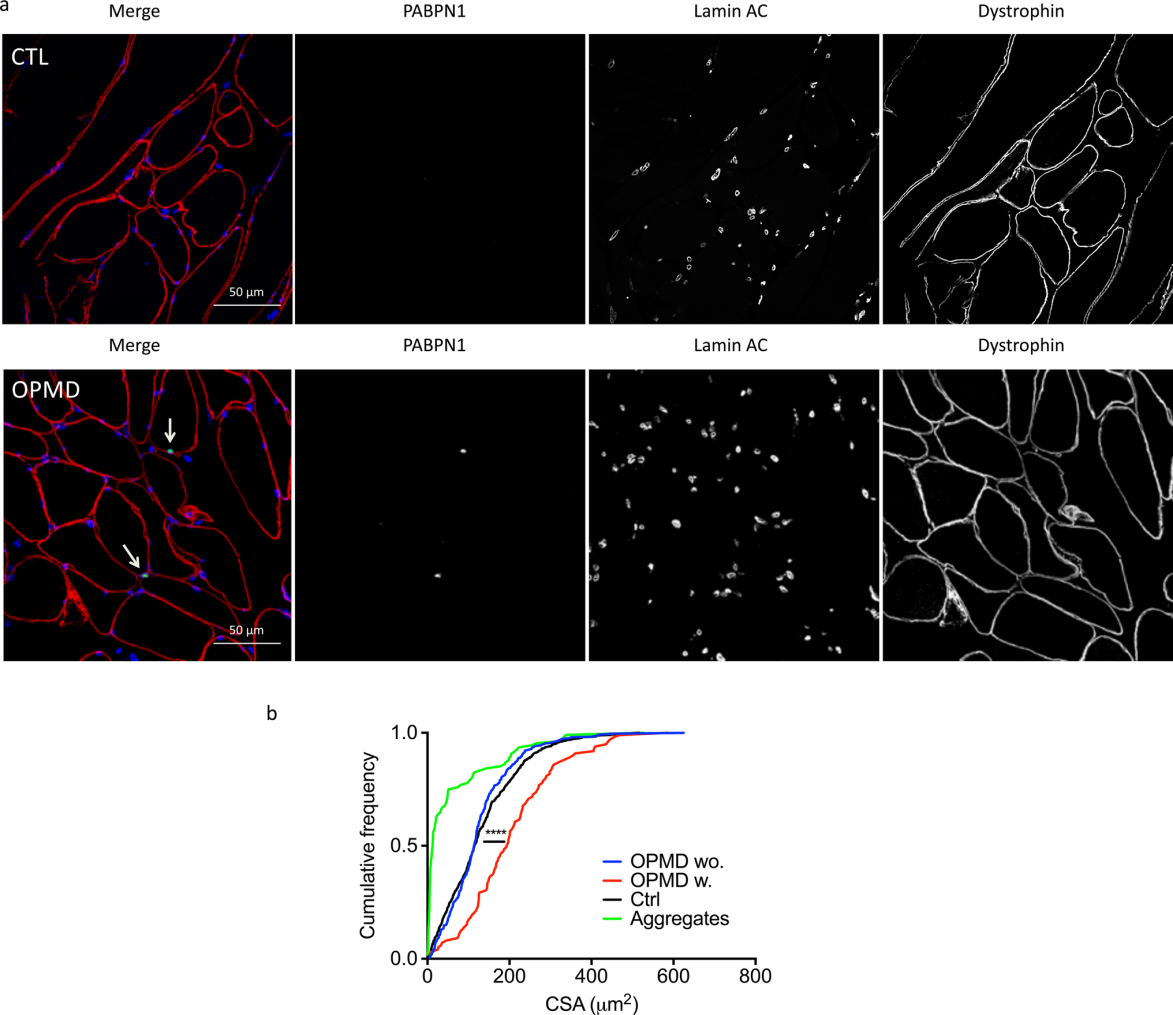
Size of myonuclei and PABPN1 aggregates. **a** PABPN1 staining (green) performed after a KCl 1 M pre-treatment on 5 μm-thick cryosections. Nuclei are counterstained with laminA/C (blue) and muscle fibers delineated with dystrophin (red) staining. Scale bar = 50 µm. Zoom-in detail and confocal images and movies are included in suppl (Fig. 4). **b** Quantification of the CSA of myonuclei and aggregates in SCM muscle biopsies from healthy (Ctrl) and OPMD subjects. OPMD wo. indicates myonuclei without aggregates and OPMD w. indicates myonuclei with aggregates. We counted at least 1000 nuclei for both Ctrl and OPMD without aggregates (OPMD wo.) and at least 80 for OPMD nuclei with aggregates (OPMD w.) combined from *n* = 4–7 biological samples. One-way Anova ****p < 0.0001 between both Ctrl or OPMD wo. and OPMD w

To gain more information on the average size of nuclear aggregates and the relative size of myonuclei with or without aggregates, we performed a laminA/C and PABPN1 immunofluorescence staining on a few biopsies from (GCN)10–13 patients and measured the CSA of both PABPN1 aggregates and myonuclei (Fig. 3a). By plotting the cumulative frequency, we observed that the CSA of aggregates was on average 10% of those of myonuclei on a transverse section and that nuclei with aggregates are larger than nuclei without (Fig. 3b). We confirmed that on a collection of 111 electron microscopy pictures from 20 patients (from A. Rouche and F. Tomé) of myonuclei containing aggregates and on these pictures, the cross-section area (CSA) of aggregates (delimited manually with a black line based on the tubulo-filamentous structure) ranged from 1 to 73% of the myonuclei’s CSA (median 13%) (Supplementary Fig. 3, online resource).

**Fig. 4:**
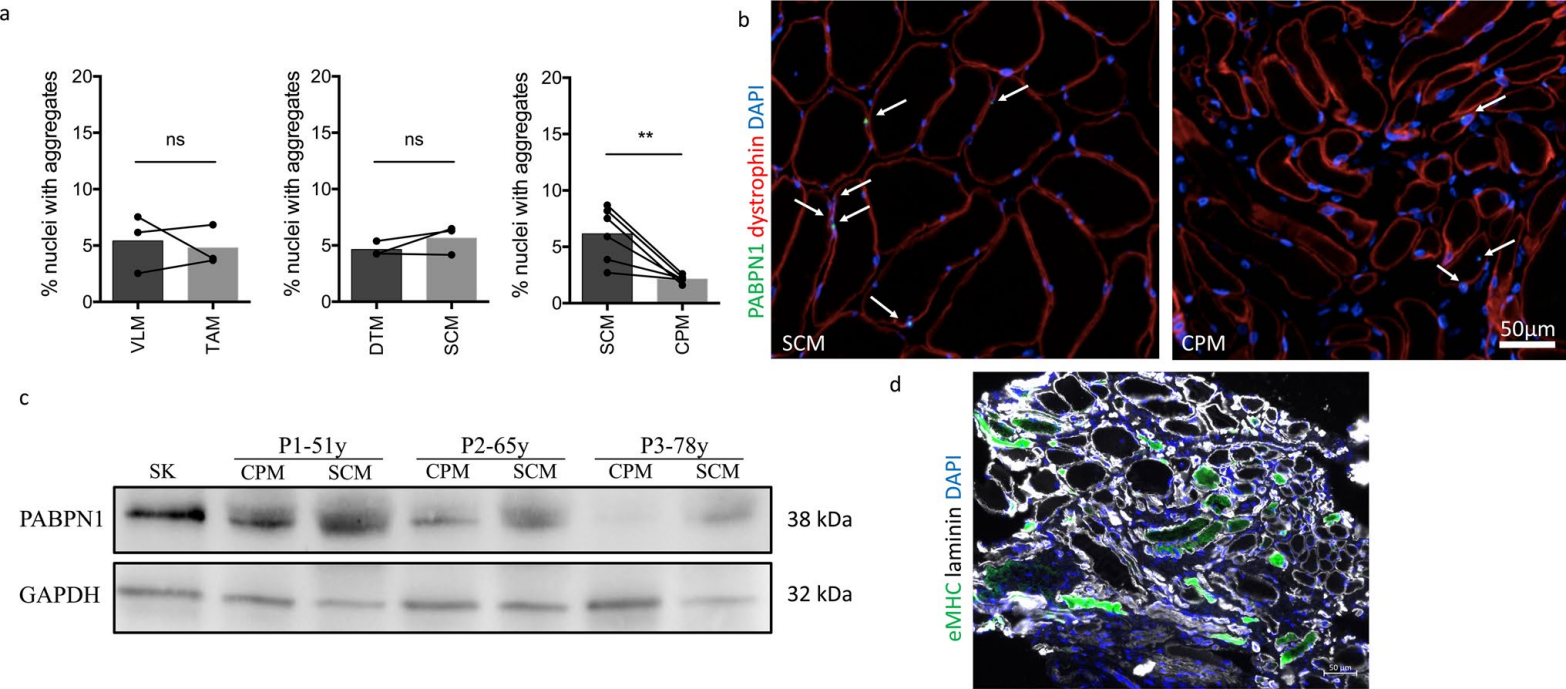
Affected CPM contains less nuclear aggregates, low level of PABPN1 and signs of regeneration. **a** Comparison of the percentage of myonuclei containing aggregates in two muscles from the same patient. *VLM* vastus lateralis muscle, *TAM* tibialis anterior muscle, *DTM* deltoid muscle, *SCM* sternocleidomastoid muscle, *CPM* cricopharyngeal muscle. Paired *t* test *ns* non-significant; **p < 0,005. **b** Muscle fibers from affected CPM are smaller than those from SCM and contain less aggregrates. PABPN1: green; dystrophin: red; dapi: blue. Scale bar = 50 µm. **c** Western-blot analysis of the level of PABPN1 in SCM and CPM from three OPMD patients. As a reference a protein extract from skin (SK) has been loaded. Western-blot is normalized with GAPDH protein levels. The age of the patient is indicated above (y: years). **d** Embryonic eMyHC immunofluorescence staining on a CPM to demonstrate regenerating fibers. eMyHC: green; laminin: white; DAPI: blue. Scale bar = 50 µm

For a restricted number of patients, we had two biopsies from two different muscles including cricopharyngeal CPM, deltoid DM, sternocleidomastoid SCM, tibialis anterior TA or vastus lateralis VLM (Table 1), the CPM being the most severely affected muscle in OPMD, leading to dysphagia. When we compared TA and VLM or DTM and SCM from the same patients, the percentage of nuclei containing aggregates was not statistically different between two distinct muscle biopsies from the same patient, regardless of their proximal or distal location. The only difference was observed when measuring the number of aggregates in the affected CPM compared to the other muscle biopsy from the same patient: less aggregates were systematically observed in CPM (Fig. 4a, b). This could be due to a lower level of PABPN1 protein in pharyngeal muscle, as previously demonstrated in mouse [5]. Indeed, on a subset of muscle biopsies, we observed a low level of PABPN1 protein in human CPM muscle samples compared to SCM (Fig. 4c). Since a loss of PABPN1 also leads to muscle degeneration, we performed an embryonic eMyHC staining (a very early marker of muscle regeneration) to evaluate the level of regeneration in these muscles. We observed many eMyHC positive fibers in OPMD CPM (Fig. 4d), suggesting on-going signs of degeneration/regeneration in this muscle, although we cannot exclude the naturally co-expression of developmental MyHC in the CPM which has a specific origin comparable to the other facial muscles [55]. We cannot exclude that the low level of aggregates is due to the loss of muscle fibers.

**Fig. 5:**
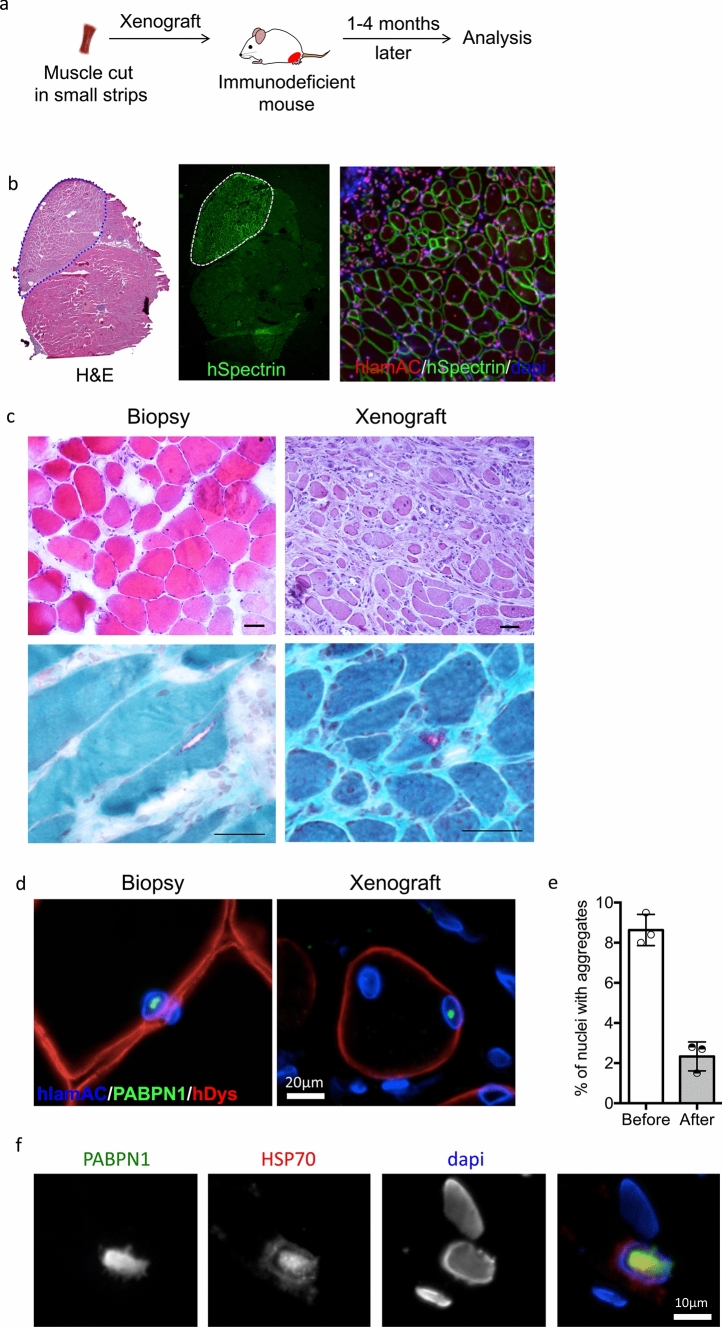
OPMD xenograft model demonstrate that nuclear PABPN1 aggregates appear soon after regeneration but at a lower amount. **a** EDL and TA from immunodeficient mice have been removed and replaced by small muscle fascicles taken from biopsy samples (SCM) of OPMD patient. Four months later, regenerated muscle can be collected and frozen for analysis. **b** Regenerated human muscle tissue (derived from the OPMD patient muscle biopsies) can be differentiated from mouse using human-specific labeling of muscle fibers (hspectrin) and muscle nuclei (hlamAC). **c** H&E and Gomori trichrome staining on the initial biopsy and on the xenografted SCM muscle highlighted features of dystrophic muscle such as centronucleated fibers and rimmed vacuoles. Scale bar = 50 µm. **d** Immunofluorescence displays nuclear aggregates on an OPMD SCM muscle biopsy prior to xenotransplantation (left) and after xenotransplantation (right). **e** Percentage of human nuclei containing aggregates before and after xenografting of three independents human OPMD SCM muscle biopsies. **f** A large nuclear aggregate containing HSP70 in the xenografted SCM muscle four months after engraftment. Scale bar = 10 µm

To further analyze the status of PABPN1 aggregates during muscle regeneration, we generated a human skeletal muscle xenograft model by transplanting fresh human OPMD muscle biopsy samples into the TA muscle bed of immunodeficient mice (Fig. 5a). In this xenograft model, the grafted human OPMD muscle first completely degenerates then regenerates (Fig. 5b). After grafting, OPMD human muscles display characteristic features of the disease, including ragged red fibers (Fig. 5c) and PABPN1 nuclear aggregates (Fig. 5d). We compared the percentage of myonuclei containing PABPN1 aggregates before (on frozen muscle biopsy) and after regeneration (on frozen xenograft several months post-surgery), and we observed an average of threefold lower amount of PABPN1-aggregate containing nuclei within the 3 xenografted biopsies compared to the initial biopsy (on average 2.3% after vs 8.6% before regeneration) (Fig. 5c, d), suggesting that the low level of PABPN1 aggregates in CPM could be due to the regeneration process. In this xenograft model, one PABPN1 aggregate was HSP70-positive (Fig. 5e).

## Discussion

Protein aggregation is the hallmark of many human neurological and neuromuscular diseases including Huntington’s disease, spinocerebellar ataxia 3 and OPMD [56]. As in many other aggregopathies, the exact contribution of PABPN1 nuclear aggregates to the human pathology in OPMD remains unclear. In an effort to investigate the exact role of PABPN1 nuclear aggregates in muscles, we collected 90 muscle biopsy samples from 73 OPMD patients of different ages and genotypes. Several studies have previously attempted to establish a correlation between the age of OPMD patients, their genotype and their phenotype but these have been limited to only a few individuals due to the rarity of the disease [3, 10, 26, 32, 39, 44, 45, 57, 67]. Recently, on a cohort of 354 French OPMD patients, we were able to demonstrate a correlation between genotype and phenotype with a more severe phenotype being observed in patients with the largest repeat expansions in the *PABPN1* gene. We also observed that patients with the largest repeats were usually diagnosed earlier than patients with short repeats, which suggests an earlier onset of disease [53]. Here using KCl pre-treatment and PABPN1 fluorescent immunostaining, we quantified the percentage and size of the nuclear aggregates within each muscle biopsy sample. Our study demonstrates a correlation between protein aggregates, age and genotype of patients using pathological human tissue. We observed an increase in the percentage of nuclei containing nuclear aggregates with age. This result is in accordance with growing evidence showing that physiological changes specific to ageing such as oxidative stress [24] or decrease in the chaperone activity [11] clearly increase protein destabilization and aggregation [7, 38]. Despite these observations, it should be noted that within one particular genotype or between patients of the same age, the percentage of nuclear aggregates is still very variable. This underlines the difficulty in establishing correlations in OPMD patients since many other factors such as genetic background or lifestyle probably play a role in the onset of the disease, on its severity and on aggregate formation and stability [22, 34]. In our study, we also observed that the composition of the nuclear aggregates varies with the genotype: (GCN)10–16 patients have more HSP70-positive aggregates (around 70% of the total number of aggregates) compared with (GCN)10–13 patients (around 30%). Differences in nuclear aggregate composition have been already suggested on a morphological basis, with some large nuclear aggregates composed of regular series of small dense masses of amorphous material interconnected with irradiating short tubular filaments and smaller aggregates classically composed of tubular filaments [12, 64]. Altogether these results suggest that nuclear aggregates are heterogeneous both in size and in composition.

HSP70 has been previously described as being included in PABPN1 nuclear aggregates, possibly through sequestration [62]. HSP70 is a molecular chaperone involved in both the refolding and handling of misfolded proteins [54] and is commonly associated with protein aggregates [29]. HSP70 binds in vitro to mutated PABPN1 with a higher affinity than to wild-type PABPN1 and it has been shown that the binding efficiency is proportional to the length of the PABPN1 expansion [62]. Wang et al*.* demonstrated that adding HSP70 to the cells reduces aggregate formation [71] and the mechanism proposed so far in the literature is that HSP70 primarily localizes to the aggregates in an attempt to prevent aggregate formation. However, if this strategy fails (eg if the nuclear level of chaperone protein is not sufficient to re-nature the amount of misfolded protein, or if the structure of the aggregates limits the accessibility and/or the efficiency of refolding), aggregates will accumulate and sequester HSP70. We hypothesize that this sequestration could lead to a loss of function of HSP70 within the cell, although this remains to be fully demonstrated. Since OPMD is a late-onset disease, it should be noted that heat shock protein levels are decreased with age [47], and our study strongly supports the following hypothetical mechanism: large expansions in PABPN1 recruit more or more rapidly HSP70 which together with a decreased efficiency of refolding with increasing age leads to a decreased bio-availability of HSP70 for the rest of the cell, altering cellular homeostasis and causing overall toxicity. PRMT1 is an arginine-methyl transferase involved in the post-translation regulation of proteins and notably important for the C-terminal methylation of PABPN1 [30]. We have also demonstrated that PRMT1 is present in all aggregates. A recent study in cancer cells demonstrated that PRMT1 directly interacts with HSP70 and catalyzes its methylation, which is then crucial for its binding and stabilization of mRNA [70]. Like HSP70, the trapping of PRMT1 in PABPN1 aggregates might also induce a loss of function, leading to reduced functional activity of this protein within muscle fibers. Interestingly, PRMT1 is also required for muscle differentiation [58] and regeneration [14] and is a key regulator of the PRMT1-FOXO3 axis. As such, it is implicated in the regulation of autophagy and muscle homeostasis [23]. A specific reduction of available PRMT1 would induce attenuation of mitochondrial biogenesis and respiratory function [58] as well as muscle atrophy [23]. Although this is not the focus of this report, further studies should investigate the PRMT1-HSP70-mRNA pathway and the effect of modulation of PRMT1 in OPMD muscles.

In agreement with previous observations in the literature [20, 64, 65], using immunofluorescence we never observed nuclear aggregates within satellite cells (data not shown), neither in muscle biopsies nor in the xenograft model. Aggregates were also rarely observed in centrally located nuclei (less than 5% of total positive myonuclei) within fibers showing that they are predominantly present in mature nuclei located at the periphery of the myofiber. In vitro characterization of myoblasts extracted from OPMD muscle failed to show nuclear aggregates either in myoblasts or myotubes. The only study describing the presence of nuclear aggregates in cultured human OPMD myotubes was when they had been co-cultured for 8-weeks with rat motoneurons [63] suggesting that maturation, a consequence of innervation and myotube contraction, is necessary to trigger nuclear aggregate formation, which occurs only in mature skeletal muscle. Interestingly, nuclear aggregates appeared in our xenograft model four months after xenotransplantation suggesting that their formation can be rapidly triggered once innervated muscle fibers reach a critical size or that innervation is necessary for aggregate formation. It has also been shown that in presymptomatic OPMD patients (patients carrying the mutation in *PABPN1* but with no clinical signs), aggregates are present in muscles before the onset of the disease [60], suggesting that their onset precedes clinical symptoms. If the pathological phenotype is due to their accumulation and/or to the increasing loss of function of proteins gradually trapped in these aggregates, decreasing or impeding the formation of nuclear aggregates should delay or prevent the onset of the disease. Indeed multiple studies have shown that decreasing the number of nuclear aggregates ameliorates the muscle phenotype in animal models [59, 62]. Although dependent on the availability of fresh human OPMD biopsies, the xenograft model will now constitute an important advance for pre-clinical studies since the use of anti-aggregate candidate molecules could be directly tested in this humanized mouse muscle to test their efficacy in a human context, providing proof of principle for clinical trials.

More importantly, it has also been proposed that the presence of PABPN1 nuclear aggregates leads to a loss of function of PABPN1 itself (similarly to the proposed model for PRMT1 and HSP70 sequestration). Several loss of function experiments either in vitro or in vivo hasclearly shown that decreasing levels of PABPN1 leads to a defect in myogenesis, and to muscle atrophy with reduced fiber cross-section area and a thickening of the extracellular matrix [50], muscle degeneration [40] and altered RNA metabolism [37, 69]. Interestingly, the level of PABPN1 protein is extremely low in skeletal muscle both in humans and in mice, as compared to other organs. In mice, it has also been shown that this level is particularly low in the pharyngeal muscles compared to other muscles [5]. For the first time, we have confirmed that PABPN1 protein level is very low in human OPMD CPM samples.

The clinically affected CPM muscles from OPMD patients are characterized by muscle fiber atrophy, high levels of fibrosis and a large number of Pax7 positive cells [31] with eMyHC positive fibers. Developmental isoforms of MHC have been observed in other facial muscles such as masseter in the absence of regeneration [17, 61]. Although we cannot exclude that this may be the case for CPM, activation and proliferation of satellite cells, and their fusion with myofibres has been described in murine extra-ocular muscles [42], but also in pharyngeal muscles [49]. This has been confirmed in human and primate extra-ocular muscles [43]. This increased activation of satellite cells has been recently proposed to be linked to the activity of FAPs secreting HGF in mice [35], which may lead to satellite cell dysfunctions and the progressive dysphagia observed both with age (achalasia) as well as in several pathologies (OPMD, IBM, DM1). The presence of a high level of fibrosis [13] may further increase this process of activation through secreted factors. However, we cannot exclude some unusual ongoing process, including fiber degeneration and regeneration, particularly in the presence of nuclear aggregates in OPMD.

In agreement with our previous study [31], we confirmed here in a larger cohort of patients that the percentage of nuclei containing aggregates is lower in OPMD CPM muscles compared to SCM muscle from the same patient. This result (more aggregates in less affected muscles) could be an argument to reinforce the decorrelation between muscle phenotype and PABPN1 aggregation but we rather propose that in the CPM the increased muscle turnover drives a decrease in aggregate counts in CPM with, as a net effect, fewer aggregates. The dual and non-exclusive models—already proposed by Apponi et al*.* [5]—of a progressive toxic role of PABPN1 nuclear aggregates leading to a loss of function—would explain why OPMD is a late onset muscular dystrophy affecting a selected set of muscles which are characterized by their low PABPN1 expression levels. Based on this hypothesis, we suggest that the addition of functional PABPN1 in the CPM muscle should be sufficient to restore muscle function and improve muscle weakness. Altogether, our data strongly support the detrimental role of nuclear aggregates in the physiopathology of the disease: by sequestering essential proteins (PABPN1, PRMT1, HSP70, RPL24, p62, GRP78/BiP…) as well as by disorganizing the nuclear space (that could, in turn, affect gene expression). Further understanding of their exact composition will be essential in the future to investigate their nature and precise mechanistic role and to help designing efficient specific therapeutic strategies. Understanding the origin, nature and role of aggregates has been a long-standing quest not only in OPMD but also in many other degenerative diseases, such as Parkinson’s disease, Huntington’s disease, and myotonic dystrophy. More generally, with the increasing lifespan of the human population, imperfections in protein homeostasis leading to misfolding and aggregation of proteins are expected to become an increasing health problem. The presence of microenvironmental stresses [28] that catalyze dysregulation of proteostasis have multiple origins and perturbed RNA metabolism (so called altered ‘ribostasis’ [48]) is one of them. Future studies on OPMD and PABPN1 will certainly help understand the more general features of RNA binding protein assembly, misfolded protein pathways, impact of ageing and/or cell type selectivity.

## Supplementary Information

Below is the link to the electronic supplementary material.

Supplementary file1 (AVI 2011 KB)

Supplementary file2 (AVI 1903 KB)

Supplementary file3 (M4V 27018 KB)

Supplementary file4 Supp Fig.1 All fibers do contain PABPN1 aggregate. a. 30 consecutive serial 5μm-cryosections of a (GCN)10-13 human OPMD SCM muscle biopsy, which represent a total volume of 1.3mm3, were cut. PABPN1 staining was performed after 1M KCl pre-treatment on these 30 sections and each section analyzed. b. Using the NanoZoomer Hamamatsu Scanner each muscle section was analyzed for aggregates. The muscle section encompasses a total of 220 fibers. Aggregates were analyzed on each fiber on a 500-μm length. c. Using Imaris software, we reconstructed the overall muscle volume to show the distribution of aggregates within the muscle (see 3D illustrative movie associated) (TIF 52934 KB)

Supplementary file5 Supp Fig.2 a. Presence of GRP78/BiP, RPL24 and p62 in PABPN1 aggregates. Scale bar=50µm (TIF 29137 KB)

Supplementary file6 Supp Fig.3 PABPN1 aggregates observed by electron microscopy. a Representative illustration of PABPN1 aggregates observed by electron microscopy. The PABPN1 aggregate is delineated with a black line. b. Distribution of aggregates size in OPMD myonuclei on a collection of 111 pictures (kind gift from F. Tome and A. Rouche). The area of the aggregate is calculated in pixels as a percentage of the total nucleus area (TIF 56485 KB)

Supplementary file7 Supp Fig.4 PABPN1 aggregates by immunofluorescence staining. PABPN1 staining (green) performed after a KCl 1M pre-treatment on 5μm-thick SCM cryosections. Nuclei are counterstained with laminA/C (blue) and muscle fibers delineated with an anti-dystrophin (red) staining. Scale bar= 50µm. Images were taken with a Confocal Spinning Disk (TIF 35237 KB)
