# Supplementary figures and images for: Assessment of PABPN1 nuclear inclusions on a large cohort of patients and in a human xenograft model of oculopharyngeal muscular dystrophy
Source: Acta Neuropathol. 2022 Oct 5;144(6):1157–70. doi: 10.1007/s00401-022-02503-7 (PMC9637588; doi:10.1007/s00401-022-02503-7)

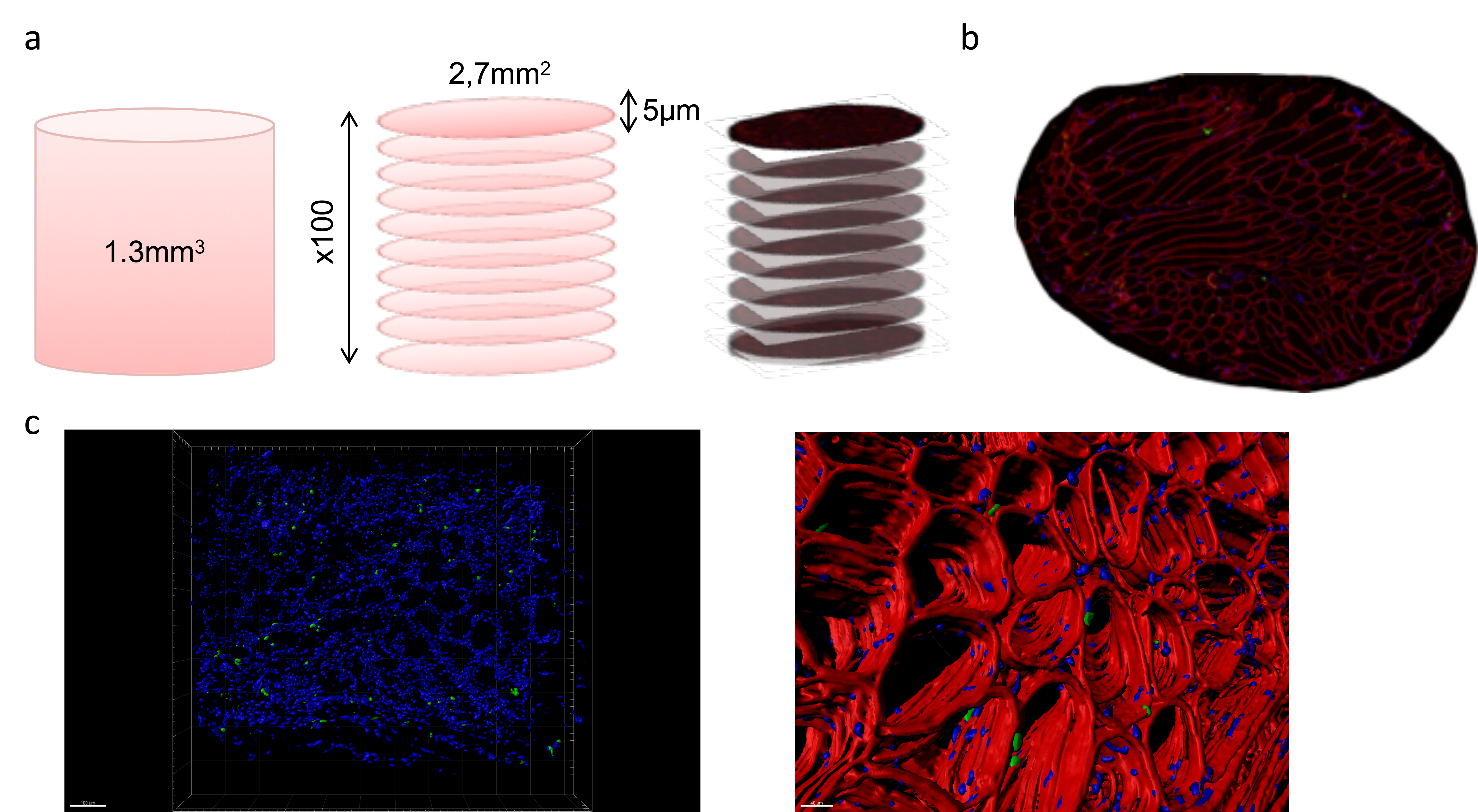

Supplement: Supplementary file 4 — Supplementary file4 Supp Fig.1 All fibers do contain PABPN1 aggregate. a. 30 consecutive serial 5μm-cryosections of a (GCN)10-13 human OPMD SCM muscle biopsy, which represent a total volume of 1.3mm3, were cut. PABPN1 staining was performed after 1M KCl pre-treatment on these 30 sections and each section analyzed. b. Using the NanoZoomer Hamamatsu Scanner each muscle section was analyzed for aggregates. The muscle section encompasses a total of 220 fibers. Aggregates were analyzed on each fiber on a 500-μm length. c. Using Imaris software, we reconstructed the overall muscle volume to show the distribution of aggregates within the muscle (see 3D illustrative movie associated) (TIF 52934 KB) [file 401_2022_2503_MOESM4_ESM.tif]

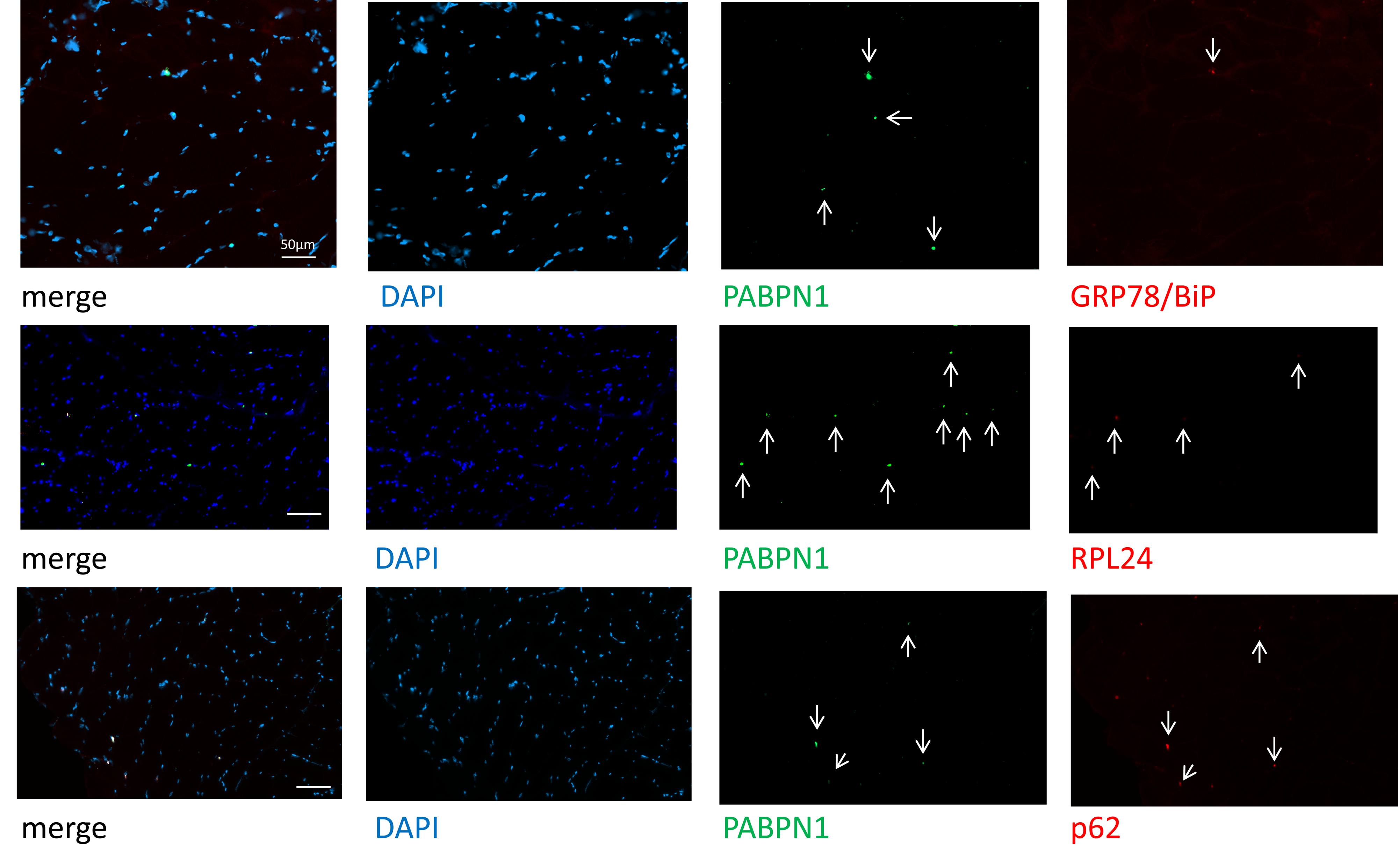

Supplement: Supplementary file 5 — Supplementary file5 Supp Fig.2 a. Presence of GRP78/BiP, RPL24 and p62 in PABPN1 aggregates. Scale bar=50µm (TIF 29137 KB) [file 401_2022_2503_MOESM5_ESM.tif]

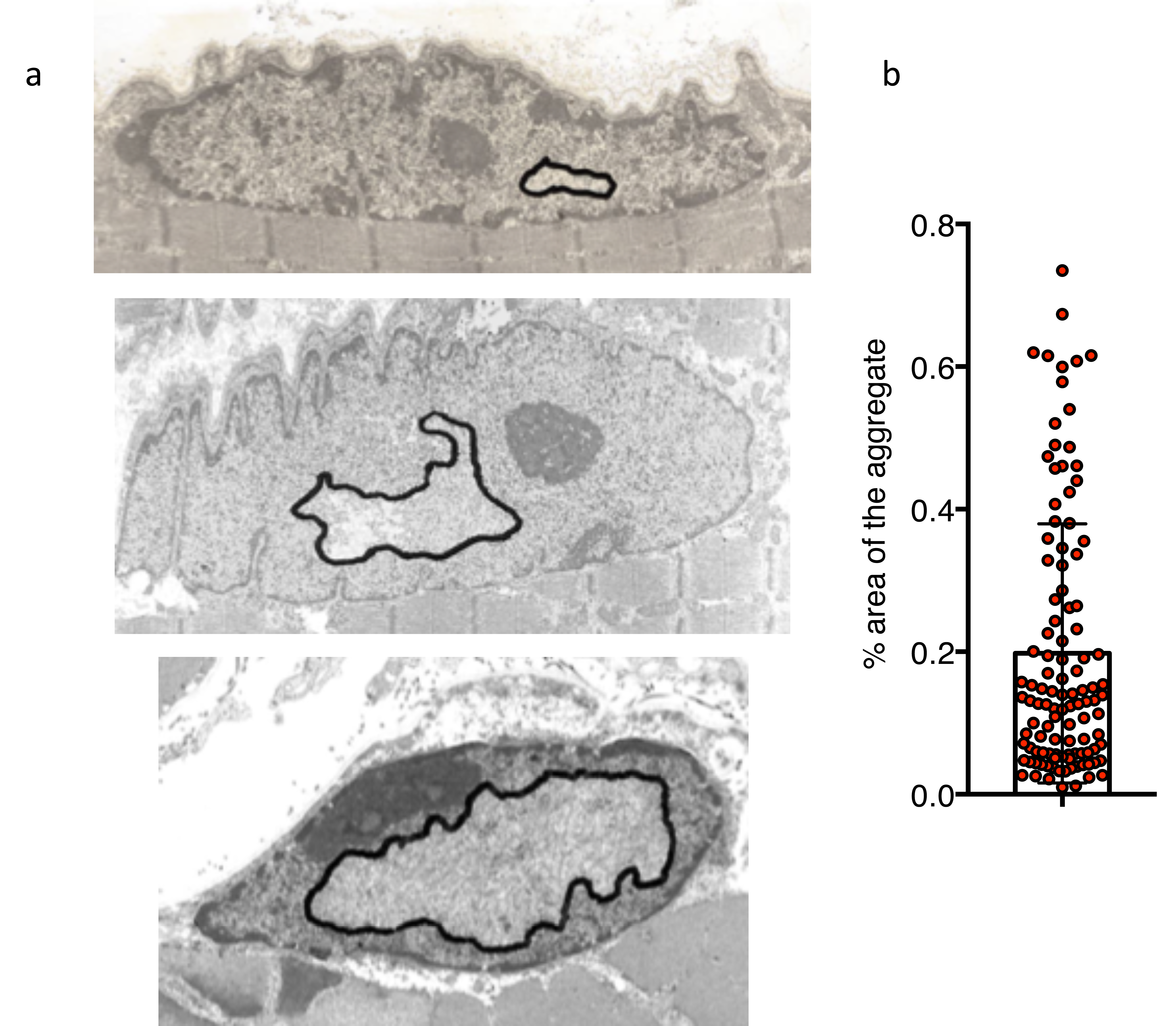

Supplement: Supplementary file 6 — Supplementary file6 Supp Fig.3 PABPN1 aggregates observed by electron microscopy. a Representative illustration of PABPN1 aggregates observed by electron microscopy. The PABPN1 aggregate is delineated with a black line. b. Distribution of aggregates size in OPMD myonuclei on a collection of 111 pictures (kind gift from F. Tome and A. Rouche). The area of the aggregate is calculated in pixels as a percentage of the total nucleus area (TIF 56485 KB) [file 401_2022_2503_MOESM6_ESM.tif]

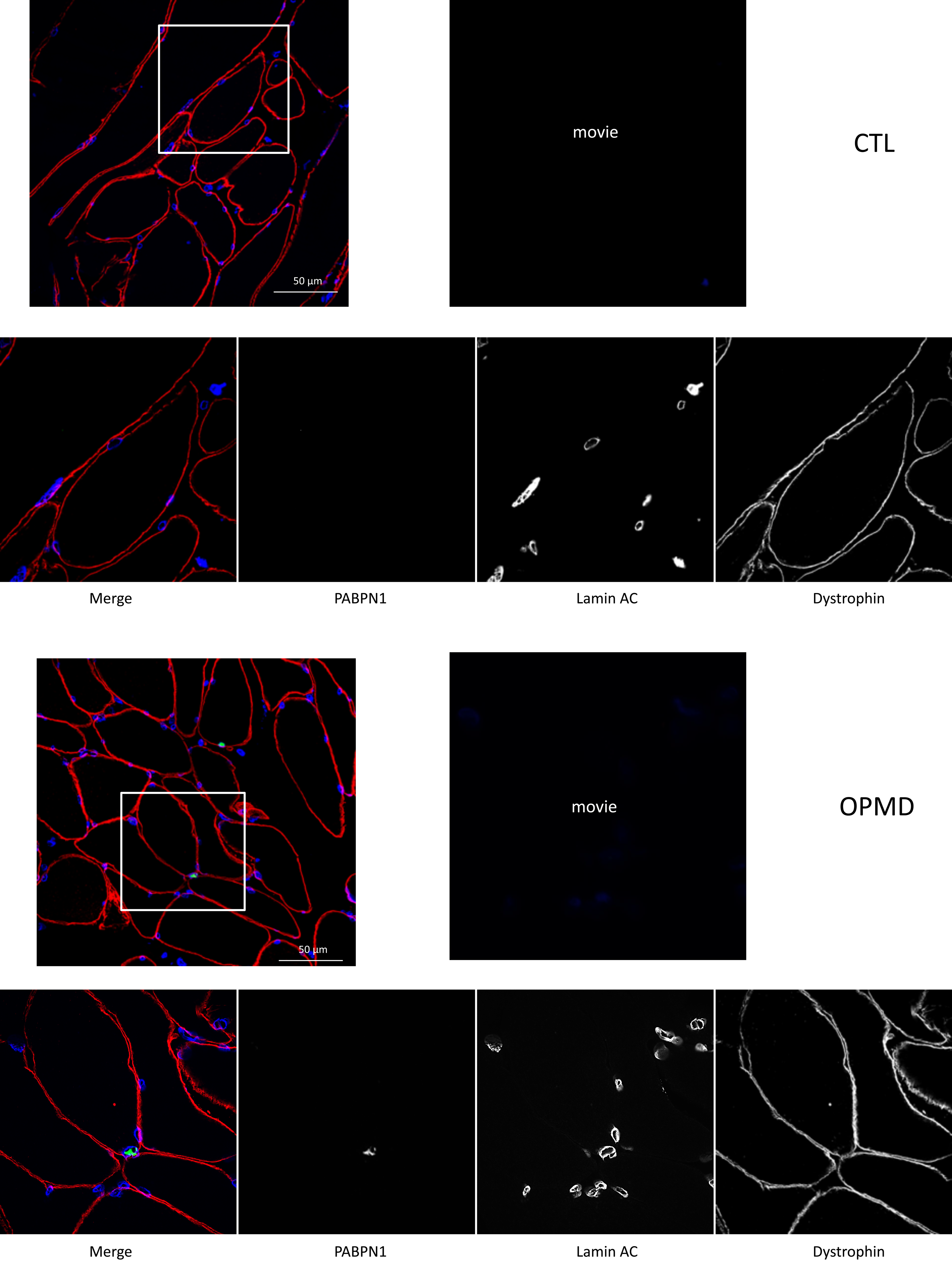

Supplement: Supplementary file 7 — Supplementary file7 Supp Fig.4 PABPN1 aggregates by immunofluorescence staining. PABPN1 staining (green) performed after a KCl 1M pre-treatment on 5μm-thick SCM cryosections. Nuclei are counterstained with laminA/C (blue) and muscle fibers delineated with an anti-dystrophin (red) staining. Scale bar= 50µm. Images were taken with a Confocal Spinning Disk (TIF 35237 KB) [file 401_2022_2503_MOESM7_ESM.tif]
